# *In Vitro* Larvicidal and Antioxidant Activity of Dihydrophenanthroline-3-carbonitriles

**DOI:** 10.1155/2014/915797

**Published:** 2014-04-24

**Authors:** A. Bharathi, Selvaraj Mohana Roopan, Abdul Abdul Rahuman, Govindasamy Rajakumar

**Affiliations:** ^1^Chemistry Research Laboratory, Organic Chemistry Division, School of Advanced Sciences, VIT University, Vellore, Tamil Nadu 632 014, India; ^2^Unit of Nanotechnology and Bioactive Natural Products, Post Graduate and Research, Department of Zoology, C. Abdul Hakeem College, Melvisharam, Vellore District, Tamil Nadu 632 509, India

## Abstract

Many naturally occurring and synthetic compounds containing dihydrocyanopyridine and cyanopyran moiety show pharmacological properties. The aim of this study is to investigate the larvicidal and antioxidant potential of dihydrophenanthroline-3-carbonitrile derivatives **4a–f**. A novel series of 2-amino-10-chloro-4,12-diphenyl-1,4,5,6-tetrahydrobenzo[*j*][1,7]phenanthroline-3-carbonitrile derivatives were synthesized by reacting different substituted acridine chalcones through Michel addition. The compounds were synthesized in excellent yields and the structures were corroborated on the basis of FT-IR, ^1^H NMR, ^13^C NMR, and ESI Mass analysis data. All the synthesized compounds were evaluated for larvicidal activity against *Aedes aegypti* and *Culex quinquefasciatus* larvae. Furthermore, the antioxidant activity was studied by the 1,1-diphenyl-2-picrylhydrazyl (DPPH) assay method. From the antioxidant assay, the compound **4c** was reported with profound antioxidant potential.

## 1. Introduction 

**Figure 1:**
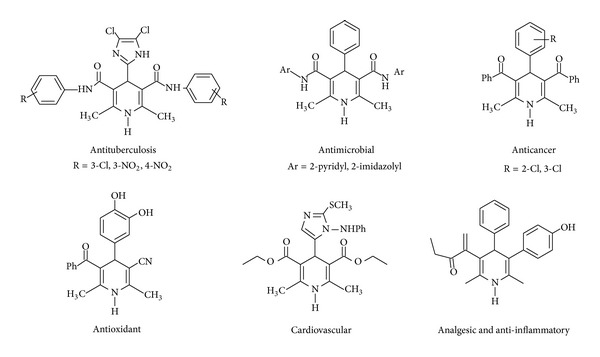
Biologically important 1,4-DHP heterocycles.

The special scopes of natural products are discovery of potential drugs with novel structures and varying biological activity. Compounds containing heterocyclic ring systems are of much importance in medicinal chemistry [1]. Because of their excellent chemotherapeutic characters, natural occurrence in living system, varied structure, and chemical properties researchers focused towards the synthesis of heterocyclic rings [2]. Among these, acridone is one of the scaffolds known to associate with several biological activities [3]. And also these acridine analogues are found in plant sources which possess various biological activities. Many plants, particularly plants pertaining to Rutaceae species, possess maximum number of acridine derivatives [4]. Dihydropyridine is an area of interest owing to the key role played in synthesis of intermediate for natural products [5] and other heterocycles. These hydropyridines are valuable synthetic intermediates and can be elaborated to the pharmacologically interesting polysubstituted piperidines and polycyclic alkaloid [6]. The reactivity of dihydropyridine is mainly involved in selective reductions [7] and electrophilic additions [8, 9] and has allowed the completion of total synthesis of alkaloids [10, 11]. Biological importance of dihydropyridines structures was elaborated in Figure 1 [12].

Vector control is facing a threat due to the emergence of resistance to synthetic insecticides. Nowadays researchers are focusing their research on a synthetic compound that kills the larvae at initial stage itself [13]. Plant extracts are acting as a potential larvicidal and antioxidant activity [14]. One of the present research interests is the synthesis of nanoparticles by biosynthetic methods. These nanoparticles are studied for larvicidal activity against various larvae [15]. All these kinds of applications are regarding the presence of various phytochemical compositions in plants. The growing interest is to synthesize heterocyclic compounds, and studying their potential uses in medicinal applications is well proved by the growing number of publications.

Taking these facts into account, our research group have been involved actively to synthesis a drug against larvae. Due to effect of synthetic compounds on larvicidal activity, our research group mainly focused on killing the larvae at initial stage itself. Earlier we reported the 7-chloro-3,4-dihydro-9-phenylacridin-1(2*H*)-one shows an effective larvicidal activity against the early fourth instar larvae of filariasis vector,* Culex quinquefasciatus,* and Japanese encephalitis vector,* Culex gelidus* (Diptera: Culicidae). The compound exhibited high larvicidal effects at 50 mg/L against both the larvae with LC_50_ values of 25.02 mg/L (*r* ^2^ = 0.998) and 26.40 mg/L (*r* ^2^ = 0.988) against* C. quinquefasciatus* and* C. gelidus*, respectively [16].

Nowadays, antioxidants that exhibit DPPH radical scavenging activity are increasingly receiving attention (Figure 3) [17]. The generation of free radicals during the metabolic process is now observed to be responsible for wide range of human diseases such as aging, cancer, atherosclerosis, arthritis, viral infection stroke, myocardial infarction, pulmonary condition, inflammatory bowel disease, and neurogenerative disease and others may be produced by reactive oxygen species [18, 19]. Antioxidants act as a major defence against radical mediated toxicity by protecting the damage caused by free radicals. Antioxidative agents are effective in the prevention and treatment of complex diseases like atherosclerosis, stroke, diabetes, Alzheimer's disease, and cancer [20, 21].

In the present study, the efforts have been laid down to synthesis of 2-amino-10-chloro-4,12-diphenyl-1,4,5,6-tetrahydrobenzo[*j*][1,7]phenanthroline-3-carbonitrile analogues,** 4a–f,** to evaluate the inherent larvicidal activity which was carried out against larva of* Aedes aegypti *and* Culex quinquefasciatus. *The model of scavenging of the stable DPPH radical is extensively used to evaluate radical scavenging activities in a very short span of time in comparison to other methods. Compound under investigation reacts with DPPH (1,1-diphenyl-picrylhydrazine) due to its hydrogen donating ability at a very rapid rate [22]. When this electron becomes paired off, the absorption decreases stoichiometrically with respect to the number of electrons taken up. Such a change in the absorbance produced in this reaction has been widely applied to test the capacity of numerous molecules to act as free radical scavengers [23]. In this way, the scavenging effects of all the synthesized compounds on the DPPH free radical were evaluated.

## 2. Materials and Methods

### 2.1. Chemistry

Melting points were measured on Elchem Microprocessor based DT apparatus using an open capillary tube and are corrected with standard benzoic acid. IR spectrum was recorded on a Perkin-Elmer spectrum RXI FT-IR spectrometer (400–4000 cm^−1^; resolution: 1 cm^−1^) using KBr pellets. ^1^H and ^13^C NMR spectra were obtained on a Bruker Avance 400 MHz spectrometer. Chemical shifts were reported on the scale relative to TMS. Exact mass measurements of the molecular ions were obtained on ESI-MS Thermo Fleet.

#### 2.1.1. General Synthesis of 2-Amino-10-choloro-4,12-diphenyl-1,4,5,6-tetrahydrobenzo[*j*][1,7]phenanthroline-3-carbonitrile Analogues ****(4a–f)****

**Scheme 1:**
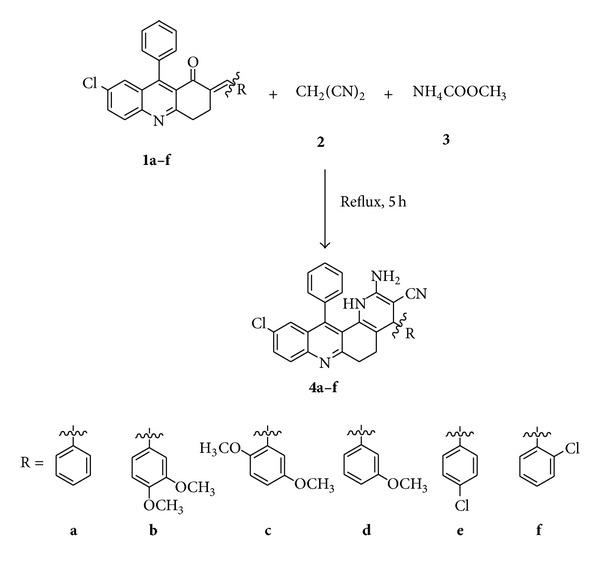
Synthesis of 2-amino-10-chloro-4,12-diphenyl-1,4,5,6-tetrahydrobenzo[*j*][1,7]phenanthroline-3-carbonitriles** (4a–f).**

(*E*)-2-(Benzylidine-7-choloro-9-phenyl3,4-dihydroacridin-1(2*H*)-ones,** 1a–f, **were synthesised by our earlier work [24]. Furthermore, synthesis of 2-amino-10-chloro-4,12-diphenyl-1,4,5,6-tetrahydrobenzo[*j*][1,7]phenanthroline-3-carbonitriles,** 4a–f**. The compound,** 1a–f** and malononitrile (0.66 g, 0.01 mol) was dissolved in absolute ethanol (15 mL). The reaction mixture was refluxed for 5 h. After completion of the reaction, the reaction mixture was cooled and poured into crushed ice. The crude products were separated by column chromatography, ethyl acetate, and petroleum ether as a solvent. All synthetic pathways were elaborated in Scheme 1 and synthesized derivatives and physical data of all synthesized compounds,** 4a–f, **were summarized in Table 1.

## 3. Biological Activity

### 3.1. Larvicidal Activity

#### 3.1.1. Insect Rearing

*A. aegypti* and* C. quinquefasciatus* larvae were collected from stagnant water area of Melvisharam (12°56′23′′ N, 79°14′23′′ E) and identified in Zonal Entomological Research Centre, Vellore (12°55′48′′ N, 79°7′48′′ E), Tamil Nadu, to start the colony, and larvae were kept in plastic and enamel trays containing tap water. They were maintained and reared in the laboratory as per the method [25].

#### 3.1.2. Larvicidal Bioassay

During preliminary screening with the laboratory trial, the larvae of* A. aegypti* and* C. quinquefasciatus* were collected from the insect-rearing cage and identified in Zonal Entomological Research Centre, Vellore. 1 mg of synthesized compounds,** 4a–f** (Table 1), was first dissolved in 100 mL of distilled water (stock solution). From the stock solution, 50 ppm was prepared with dechlorinated tap water. DMSO (Qualigens) was used as an emulsifier at the concentration of 2.0% in the final test solution. The larvicidal activity was assessed by the procedure of WHO 1996 with some modification. For bioassay test, larvae were taken in five batches of 20 in 249 mL of water and 1 mL of the desired synthetic compounds,** 4a–f,** at different concentrations (3.12 to 50 ppm). The control was set up with DMSO 2.0% and dechlorinated tap water. The numbers of dead larvae were counted after 24 h of exposure, and the percentage of mortality was reported from the average of three replicates. The experimental media in which 100% mortality of larvae occurs alone were selected for dose-response bioassay.

#### 3.1.3. Dose-Response Bioassay

From the stock solution, different concentrations ranging from 3.12 to 50 ppm were prepared for larvicidal activity. Based on the preliminary screening results, synthetic compounds,** 4a–f,** were subjected to dose-response bioassay for larvicidal activity against the larvae of* A. aegypti* and* C. quinquefasciatus*. The numbers of dead larvae were counted after 24 h of exposure, and the percentage of mortality was reported from the average of three replicates. However, at the end of 24 h, the selected test samples turned out to be equal in their toxic potential.

#### 3.1.4. Statistical Analysis

The average larval mortality data were subjected to probit analysis for calculating LC_50_, LC_90_, and other statistics at 95% fiducial limits of upper confidence limit and lower confidence limit, and chi-square values were calculated using the software [26]. Results with *P* < 0.05 were considered to be statistically significant.

### 3.2. Antioxidant Activity

All the synthesized compounds,** 4a–f,** were to be examined for antioxidant activity. Antioxidant assay [27] is based on the measurements of the scavenging ability of compounds towards the stable 2,2-diphenyl-1-picrylhydrazyl radical (DPPH). The disappearance of this commercially available radical is measured spectrophotometrically at 517 nm in an ethanolic solution. The antioxidant activity was expressed as the 50% inhibitory concentration (IC_50_) based on the amount of compound required for a 50% decrease of the initial DPPH radical concentration.

**Figure 2:**
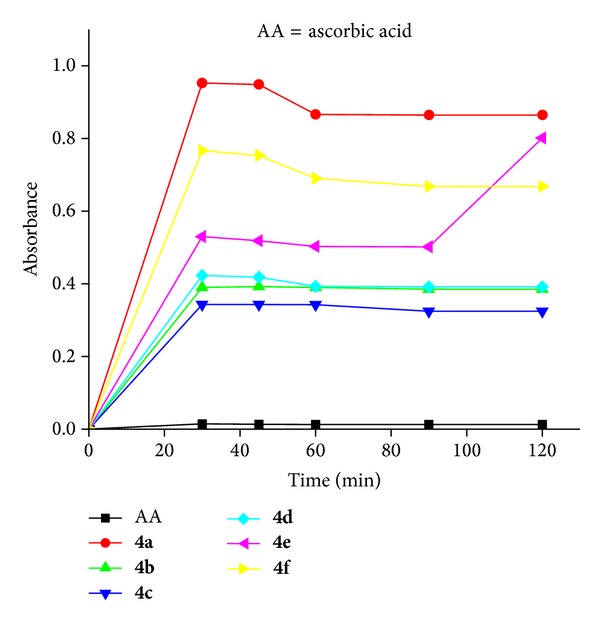
DPPH antiradical scavenging activity with time-dependent antiradical scavenging activity.

**Figure 3:**
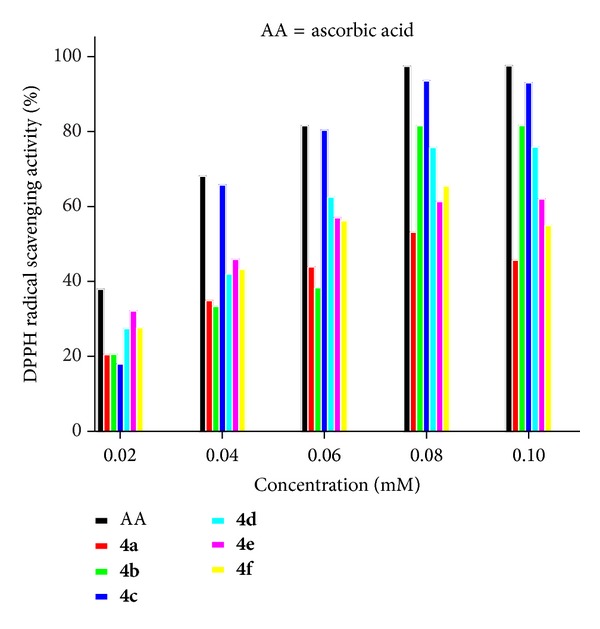
Percentage radical scavenging activity at various concentrations.

DPPH antiradical scavenging activity was also time dependent. The radical scavenging activity of the tested samples, expressed as percentage inhibition of DPPH, was calculated according to the following formula:

(1) $$\begin{array}{c} \text{IC}\left(\%\right)=\left[\frac{A_{0}-A_{t}}{A_{0}}\right]\times 100, \end{array}$$

where *A*_*t*_ is the absorbance value of the tested sample and *A*_0_ is the absorbance value of blank sample at a particular time. The data for antioxidation is presented as means ± SD of three determinations. The synthesized compounds used for antioxidant assay are of 1 mM concentration. Absorbance taken after 30, 45, 60, 90, and 120 min was plotted against absorption (Figure 1). Percentage inhibition was plotted against various concentrations 0.001, 0.002, 0.004, 0.006, 0.008, and 0.1 (Figure 2); the linear regression analysis equation was used to obtain the IC_50_ value. A lower IC_50_ value indicates greater antioxidant activity.

## 4. Spectral Details of Synthesized Compounds (4a–f)

### 4.1. 2-Amino-10-chloro-4,12-diphenyl-1,4,5,6-tetrahydrobenzo[*j*][1,7]phenanthroline-3-carbonitrile **(4a)**

Pale yellow solid; M.F: C_29_H_21_ClN_4_; Yield 79%; M.P: 144–146°C; FT-IR (KBr) *ν*_max⁡_ (cm^−1^): 3444 (–NH_2_), 3236 (–NH_sym_), 2222 (–C=N); ^1^H NMR (CDCl_3_): *δ* (ppm), 2.17–2.22 (*t*, *J* = 10.8 Hz, 1H, –CH_2_), 2.40–2.44 (*t*, *J* = 7.8 Hz, 1H, –CH_2_), 3.00–3.04 (*t*, *J* = 8 Hz, 1H, –CH_2_), 3.13–3.17 (*t*, *J* = 8 Hz, 1H, –CH_2_), 3.41 (*s*, 2H, –NH_2_) 3.45 (*s*, H, –CH), 4.04 (*s*, 1H, –NH), 7.23–7.25 (*m*, 4H), 7.32–7.39 (*m*, 4H), 7.53–7.59 (*m*, 4H), 7.92–7.94 (*d*, *J* = 8.4 Hz, 1H); ^13^C NMR (CDCl_3_): *δ* (ppm), 24.0, 32.8, 43.2, 60.1, 117.9, 119.4, 120.7, 125.5, 127.6, 127.8, 128.0, 128.1, 128.2, 128.5, 129.0, 129.1, 129.2, 130.3, 130.4, 132.4, 138.7, 140.4, 140.9, 142.5, 145.0, 158.1, 158.5; Exact Mass: 460.15; Found ESI-MS *m*/*z*: 462.29 [*M* + 2].

### 4.2. 2-Amino-10-chloro-4-(3,4-dimethoxyphenyl)-12-phenyl-1,4,5,6-tetrahydro benzo[*j*][1,7]phenanthroline-3-carbonitrile  **(4b)**

Yellow solid; M.F: C_31_H_25_ClN_4_O_2_; Yield 80%; M.P: 230–232°C; FT-IR (KBr) *ν*_max⁡_ (cm^−1^): 3450 (–NH_2_), 3330 (–NH_sym_), 2192 (–C=N); ^1^H NMR (DMSO-d_6_): *δ* (ppm), 2.04–2.12 (*m*, 1H, –CH_2_), 2.34–2.40 (*m*, 1H, CH_2_), 2.91–2.97 (*m*, 1H, –CH_2_), 3.06–3.12 (*m*, 1H, –CH_2_), 3.45 (*s*, 2H, NH_2_), 3.73-3.74 (*d*, *J* = 1.2 Hz, 6H, 2–OCH_3_) 4.10 (*s*, 1H, –CH), 4.90 (*s*, 1H, –NH), 6.74–6.76 (*d*, *J* = 7.6 Hz, 2H), 6.88–6.94 (*d*, *J* = 8 Hz, 1H), 7.23-7.24 (*d*, *J* = 2.4 Hz, 1H), 7.35–7.41 (*m*, 2H), 7.54–7.59 (*m*, 3H), 7.71–7.75 (dd, *J* = 2.4 Hz, *J* = 7.2 Hz, 1H), 7.91–7.99 (*d*, *J* = 8.8 Hz, 1H); ^13^C NMR (DMSO-d_6_): *δ* (ppm), 23.9, 32.8, 42.7, 55.9, 60.2, 110.9, 117.2, 117.9, 119.3, 120.2, 120.6, 125.3, 127.5, 127.9, 128.1, 128.4, 128.9, 129.0, 130.1, 130.3, 132.3, 135.0, 138.5, 140.3, 140.7, 144.9, 148.6, 149.3, 157.8, 158.4; Exact Mass: 520.17; Found ESI-MS *m*/*z*: 522.1 [*M* + 2].

### 4.3. 2-Amino-10-chloro-4-(2,5-dimethoxyphenyl)-12-phenyl-1,4,5,6-tetrahydrobenzo[*j*][1,7]phenanthroline-3-carbonitrile  **(4c)**

White solid; M.F: C_31_H_25_ClN_4_O_2_; Yield 76%; M.P: 238–240°C; FT-IR (KBr) *ν*_max⁡_ (cm^−1^): 3452 (–NH_2_), 3329 (–NH_sym_), 2198 (–C=N); ^1^H NMR (DMSO-d_6_): *δ* (ppm), 2.04–2.11 (*m*, 1H, –CH_2_), 2.33–2.39 (*m*, 1H, –CH_2_), 2.88–2.96 (*m*, 1H, –CH_2_), 3.07–3.14 (*m*, 1H, –CH_2_) 3.33 (*s*, 2H, –NH_2_), 3.68–3.72 (*d*, *J* = 15.8 Hz, 6H, 2-OCH_3_) 4.49 (*s*, 1H, –CH), 4.86 (*s*, 1H, –NH), 6.63 (*d*, *J* = 3.2 Hz, 1H), 6.80–6.83 (dd, *J* = 3.2 Hz, *J* = 2.8 Hz, 1H), 6.95–6.98 (*d*, *J*= 9.2 Hz, 1H), 7.20-7.21 (*d*, *J* = 2.4 Hz, 1H), 7.36-7.37 (*d*, *J* = 3.2 Hz, 2H), 7.54–7.60 (*m*, 3H), 7.71–7.73 (dd, *J* = 2 Hz, 2 Hz, 1H), 7.97–7.99 (*d*, *J* = 8.8 Hz, 1H); ^13^C NMR (DMSO-d_6_): *δ* (ppm), 23.9, 32.8, 42.7, 55.9, 60.2, 110.9, 117.2, 117.9, 119.3, 120.2, 120.6, 125.3, 127.5, 127.9, 128.1, 128.4, 128.9, 129.0, 130.1, 130.3, 132.3, 135.0, 138.5, 140.3, 140.7, 144.9, 148.6, 149.3, 157.8, 158.4; Exact Mass: 520.17; Found ESI-MS *m*/*z*: 522.1 [*M* + 2].

### 4.4. 2-Amino-10-chloro-4-(3-methoxyphenyl)-12-phenyl-1,4,5,6-tetrahydrobenzo[*j*][1,7]phenanthroline-3-carbonitrile  **(4d)**

White solid; M.F: C_30_H_23_ClN_4_O; Yield 80%; M.P: 140–142°C; FT-IR (KBr) *ν*_max⁡_ (cm^−1^): 3446 (–NH_2_), 3325 (–NH_sym_), 2193 (–C=N); ^1^H NMR (DMSO-d_6_): *δ* (ppm), 2.03–2.11 (*m*, 1H, –CH_2_), 2.35–2.43 (*m*, 1H, –CH_2_), 2.89–2.96 (*m*, 1H, –CH_2_), 3.08–3.14 (*m*, 1H, –CH_2_), 3,33 (*s*, 2H, –NH_2_), 3.74 (*s*, 3H, –OCH_3_), 4.11 (*s*, 1H, –CH), 4.93 (*s*, 1H, –NH), 6.77–6.95 (*m*, 3H), 7.21-7.22 (*d*, *J* = 2.4 Hz, 2H), 7.25–7.30 (*t*, *J* = 7.8 Hz, 1H), 7.35–7.39 (*t*, *J* = 8 Hz, 1H), 7.56–7.58 (*m*, 3H), 7.70–7.73 (dd, *J* = 2.4 Hz, *J* = 2 Hz, 1H), 7.96–7.98 (*d*, *J* = 8.8 Hz 1H); ^13^C NMR (DMSO-d_6_): *δ* (ppm), 23.5, 32.0, 42.4, 54.9, 57.0 112.4, 113.5, 118.0, 119.5, 119.8, 120.5, 124.4, 127.6, 127.8, 128.0, 128.1, 128.5, 128.7, 129.7, 129.8, 130.5, 132.8, 137.4, 139.6, 139.9, 144.2, 144.8, 158.1, 158.8, 159.4; Exact Mass: 490.16; Found ESI-MS *m*/*z*: 494.0 [*M* + 4].

### 4.5. 2-Amino-10-chloro-4-(2-chlorophenyl)-12-phenyl-1,4,5,6-tetrahydrobenzo[*j*][1,7]phenanthroline-3-carbonitrile  **(4e)**

Orange solid; M.F: C_29_H_20_Cl_2_N_4_; Yield 75%; M.P: 210–212°C; FT-IR (KBr) *ν*_max⁡_ (cm^−1^): 3446 (–NH_2_), 3322 (–NH_sym_), 2191 (–C=N); ^1^H NMR (DMSO-d_6_): *δ* (ppm), 2.13–2.21 (*m*, 1H, –CH_2_), 2.37–2.43 (*m*, 1H, –CH_2_), 2.97–3.06 (*m*, 1H, –CH_2_), 3.13–3.20 (*m*, 1H, –CH_2_), 3.38 (*s*, 1H, –CH), 4.69 (*s*, 1H, –NH), 5.00 (*s*, 2H, –NH_2_), 7.29-7.30 (*d*, *J* = 2 Hz, 1H), 7.32–7.36 (*d*, *J* = 6.8 Hz, 3H), 7.40-7.41 (*d*, *J* = 1.2 Hz, 1H), 7.44 (*s*, 1H), 7.46-7.47 (*d*, *J* = 1.6 Hz, 1H), 7.55–7.59 (*m*, 3H), 7.72–7.74 (dd, *J* = 2.4 Hz, *J* = 2.4 Hz, 1H), 7.97–7.99 (*d*, *J* = 8.8 Hz, 1H); ^13^C NMR (DMSO-d_6_): *δ* (ppm), 23.8, 32.6, 42.5, 59.5, 117.1, 119.0, 120.3, 125.4, 127.5, 128.0, 128.1, 128.4, 128.9, 129.0, 129.1, 129.2, 130.1, 130.4, 132.3, 133.6, 138.5, 140.5, 140.9, 141.0, 144.9, 158.0, 158.2; Exact Mass: 494.11; Found ESI-MS *m*/*z*: 496.1 [*M* + 2].

### 4.6. 2-Amino-10-chloro-4-(4-chlorophenyl)-12-phenyl-1,4,5,6-tetrahydrobenzo[*j*][1,7]phenanthroline-3-carbonitrile  **(4f)**

White solid; M.F: C_29_H_20_Cl_2_N_4_; Yield 71%; M.P: 255–257°C; FT-IR (KBr) *ν*_max⁡_ (cm^−1^): 3442 (–NH_2_), 3315 (–NH_sym_), 2205 (–C=N); ^1^H NMR (DMSO-d_6_): *δ* (ppm), 1.95–2.03 (*m*, 1H, –CH_2_), 2.33–2.41 (*m*, 1H, –CH_2_), 2.51 (*s*, 2H, –NH_2_), 2.89–2.96 (*m*, 1H, –CH_2_), 3.08–3.15 (*m*, 1H, –CH_2_), 3.38 (*s*, 1H, –CH), 4.69 (*s*, 1H, –NH), 5.00 (*s*, 2H, –NH_2_), 7.23-7.24 (*d*, *J* = 2 Hz, 1H), 7.29–7.33 (*m*, 2H), 7.36–7.38 (*d*, *J* = 6.8 Hz, 2H). 7.44 (*s*, 1H), 7.45-7.46 (*s*, 1H), 7.46 (*d*, *J* = 1.6 Hz, 1H), 7.54–7.62 (*m*, 3H), 7.72–7.74 (dd, *J* = 2.4 Hz, *J* = 2.4 Hz, 1H), 7.97–7.99 (*d*, *J* = 8.8 Hz, 1H); ^13^C NMR (DMSO-d_6_): *δ* (ppm), 23.4, 31.9, 55.5, 116.7, 119.2, 120.4, 124.5, 127.7, 128.0, 128.1, 128.7, 129.1, 129.7, 129.8, 130.5, 130.8, 130.9, 132.4, 137.3, 139.7, 140.5, 144.3, 158.6, 158.8; Exact Mass: 494.11; Found ESI-MS *m*/*z*: 496.1 [*M* + 2].

## 5. Result and Discussion 

Results on the larvicidal activities of** 4a–f** obtained in this study that were summarized in Table 2 confirm their potential for the control of larval population of vectors. Compounds** 4a**,** 4c**, and** 4d** resulted in moderate mortality against* A. aegypti* 22.4, 72.6, and 52.6% and against* C. quinquefasciatus* 36.2, 87.0, and 46.2%; however, the highest larval mortality was observed in** 4b**,** 4e**, and** 4f** against* A. aegypti* 78.2, 100.0, and 82.0 and against* C. Quinquefasciatus* 92.8, 100.0, and 76.6 at 50 ppm. LC50 and LC90 values are calculated for active compounds. The fourth instar larvae of* A. aegypti* had values of LC_50_ 103.40, 69.94, and 158.98 and LC_90_ 424.12, 195.70, and 445.78 for** 4b**,** 4e**, and** 4f,** respectively, and larvae of* C. Quinquefasciatus* had values of LC_50_ 184.24, 82.29, and 142.22 and LC_90_ 643.29, 272.36, and 372.34 for compounds** 4b**,** 4e,** and** 4f,** respectively, in Table 3. The *χ* ^2^ values are significant at *P* < 0.05 level. The 95% confidence limits LC_50_ (LCL–UCL) and LC_90_ (LCL–UCL) were also calculated. Larval mortality was observed after 24 h exposure; no mortality was observed in the control group. The results of larvicidal activity clearly indicate that the percentage of mortality is directly proportional to the concentration of the compounds. Synthesized compounds,** 4a–f**, were used at different concentrations, ranging from 3.12 to 50, respectively.

The entire synthesized compounds scavenged DPPH radical significantly in a concentration-dependent manner. Their comparable scavenging activities were expressed in IC_50_ (concentration required for 50% inhibition of l M DPPH concentration) value. As presented in Table 4, compound** 4c** with IC_50_ values in the range of 41 *μ*M showed good radical scavenging activities in comparison with ascorbic acid which was attributed to the presence of two methoxy aryl group of** 4c** (that can donate hydrogen atoms) and governs the main factor behind their ability to be scavenged by DPPH. After donating a hydrogen atom, compounds exist in their radical form, and the electron conjugation effect in the structure stabilizes the radical of DPPH.

The difference in activity amongst compounds** 4a–f** was due to the difference in the substitution of these compounds. Amongst them, compounds** 4b** and** 4c** with two methoxy substituents showed higher hydrogen donor ability to DPPH radical. Compounds** 4b** and** 4c** IC_50_ values were 54 and 41, respectively, since the corresponding IC_50_ values for all synthesized compounds were higher than compounds** 4b** and** 4c**. Compound** 4c** has two methoxy groups which are para to each other. Therefore, radical ion resulting from the abstraction of –H atom by DPPH would stabilize by other hydroxyl groups. While in compound** 4b** both the methoxy groups are in meta and para positions, the radical ion resulted from the abstraction of H atom not that much stabilized from methoxy group compared with** 4c**. Thus, compound** 4d** shows better scavenging effect compared to compounds** 4e **and** 4f** due to the presence of single methoxy group. Compounds** 4a**,** 4e,** and** 4f **are less active compared with other derivativesbecause of the absence of any electron releasing group. The results suggest that our compound possesses less activity when compared to 9-aminoacridine propranolol (IC_50_ = 13.6) [28].

## 6. Conclusion

Our study demonstrated the novel 2-amino-10-chloro-4,12-diphenyl-1,4,5,6-tetrahydrobenzo[*j*][1,7]phenanthroline-3-carbonitriles** 4a–f **were synthesized from (*E*)-2-(3,4-dimethoxybenzylidene)-7-choloro-3,4-dihydro-9-phenylacridin-1-(2*H*)-ones** 1a–f**. The synthesized compounds,** 4a–f,** were used as vector control agents:* Aedes aegypti *and* Culex quinquefasciatus* larvae. These encouraging results of the present study provide useful information for further structural optimization of these compounds and a rapid detection for the activity of the target compounds. Furthermore, DPPH radical scavenging activities are evaluated on all synthesized compounds. From the antioxidant results synthesized compound** 4c **shows higher scavenging activity among all other derivatives.

## Figures and Tables

**Table 1:** Summary of synthesized dihydropyridine derivatives (**4a–f**) via Scheme 1.

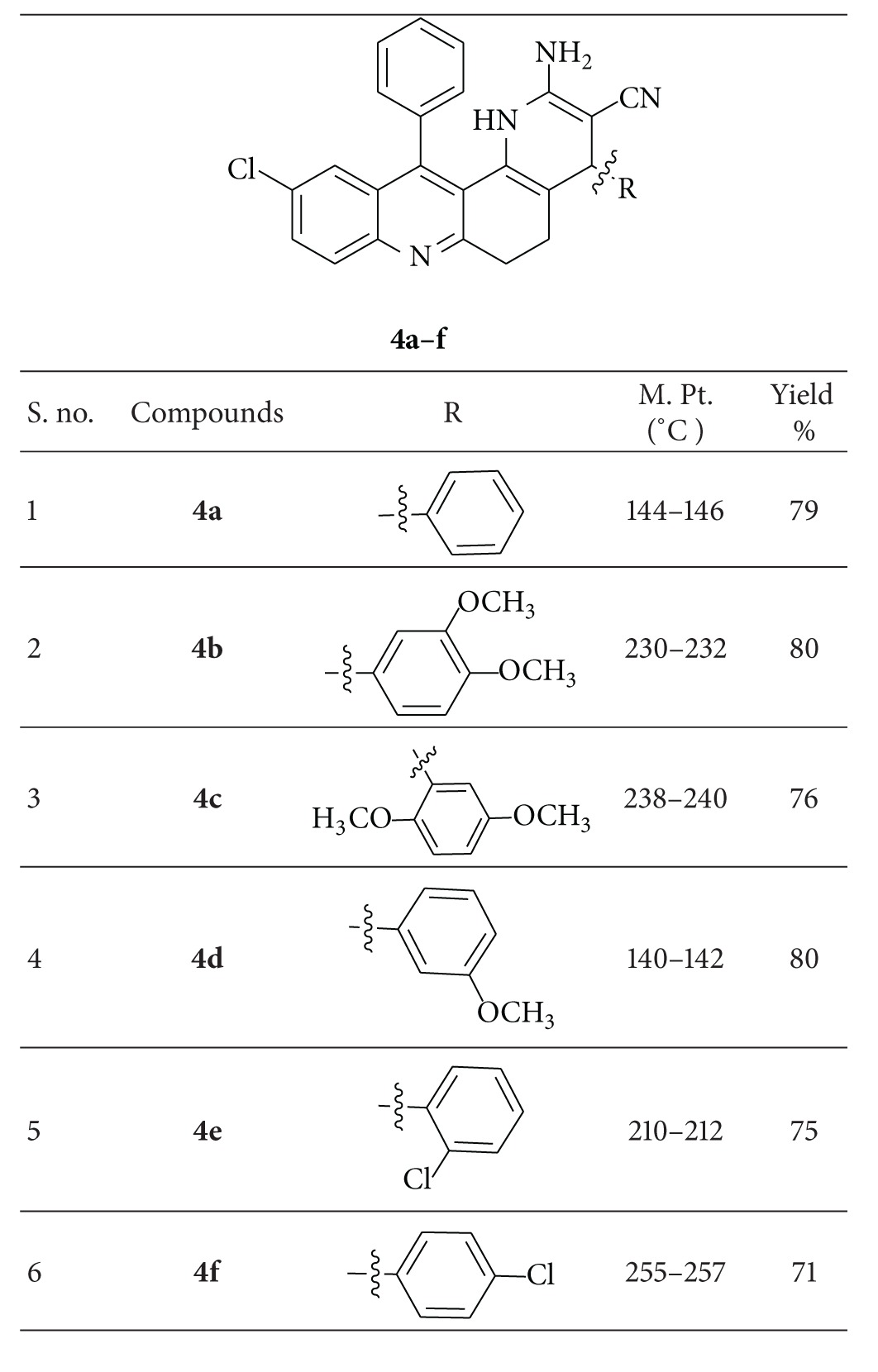

**Table 2:** Larvicidal activity, mean efficacy (percentage ± S.D) of synthetic compounds against fourth instar larvae of *Aedes aegypti *and *Culex quinquefasciatus*.

Compound	Concentration (ppm)	% Mortality ^a^(ppm) ± SD	
		*A. aegypti *	*C. quinquefasciatus *
**4a**	50	22.4 ± 6.69	36.2 ± 4.24
	25	16.2 ± 1.26	16.2 ± 6.00
	12.5	12.0 ± 4.01	10.0 ± 4.28
	6.25	8.2 ± 0.24	6.8 ± 0.43
	3.12	4.6 ± 0.62	2.0 ± 1.86

**4b**	50	78.2 ± 5.72	92.8 ± 8.18
	25	64.8 ± 1.79	71.4 ± 7.70
	12.5	55.4 ± 8.38	58.0 ± 7.91
	6.25	42.2 ± 1.39	36.2 ± 8.43
	3.12	22.4 ± 6.69	46.8 ± 5.67

**4c**	50	72.6 ± 6.05	87.0 ± 3.39
	25	68.6 ± 2.15	54.4 ± 2.04
	12.5	45.2 ± 8.83	34.8 ± 2.26
	6.25	24.2 ± 4.06	10.2 ± 3.42
	3.12	14.6 ± 2.84	8.0 ± 1.53

**4d**	50	52.6 ± 2.48	46.2 ± 2.84
	25	48.4 ± 2.68	43.2 ± 0.66
	12.5	39.0 ± 6.73	65.0 ± 7.65
	6.25	26.4 ± 10.56	39.4 ± 14.25
	3.12	18.6 ± 3.60	22.6 ± 3.45

**4e**	50	100 ± 0.00	100 ± 0.00
	25	84.0 ± 1.79	91.4 ± 2.80
	12.5	65.4 ± 2.38	58.0 ± 6.90
	6.25	48.2 ± 4.04	42.2 ± 2.06
	3.12	26.4 ± 2.06	36.8 ± 5.04

**4f**	50	82.4 ± 0.62	76.6 ± 1.04
	25	66.1 ± 1.96	48.9 ± 1.43
	12.5	52.0 ± 1.49	36.2 ± 0.18
	6.25	28.2 ± 0.24	28.1 ± 0.22
	3.12	16.8 ± 1.22	18.0 ± 1.16

**Table 3:** LC_50_, LC_90_, and other statistical analysis of synthetic compounds against fourth instar larvae of *Aedes aegypti* and *Culex quinquefasciatus*.

Sample code	Species	LC_50_ ± SE (ppm)	UCL–LCL	LC_90_ ± SE (ppm)	UCL–LCL	*χ* ^2^ (df = 4)
**4b **	*A. aegypti *	103.40 ± 7.13	128.48–82.30	424.12 ± 49.96	660.92–464.35	10.64
	*C. quinquefasciatus *	184.24 ± 6.04	198.02–124.16	643.29 ± 62.28	840.26–629.06	12.88

**4e**	*A. aegypti *	69.94 ± 2.56	48.89–35.07	195.70 ± 24.30	214.73–147.66	10.56
	*C. quinquefasciatus *	82.29 ± 2.74	64.77–46.81	272.36 ± 38.21	238.03–166.62	12.80

**4f**	*A. aegypti *	158.98 ± 2.54	124.89–115.07	445.78 ± 24.38	273.78–227.64	10.24
	*C. quinquefasciatus *	142.22 ± 2.74	87.73–46.82	372.34 ± 18.21	248.03–186.12	8.82

Control: nil mortality; LC_50_: lethal concentration that kills 50% of the exposed larvae; LC_90_: lethal concentration that kills 90% of the exposed larvae; UCL: upper confidence limit; LCL: lower confidence limit; *χ* ^2^: chi–square; df: degree of freedom significant at *P* < 0.05 level.

**Table 4:** 50% inhibition of scavenging activity for compounds **4a–f**.

Compounds	^ a^IC_50_ × 10^−3^
**Ascorbic acid**	38
** 4a**	53
** 4b**	54
** 4c**	41
** 4d**	68
** 4e**	71
** 4f**	80
9-Amino-acridine-propranolol	13.6

^a^IC_50_ values were determined by linear regression analysis using different concentrations in triplicate.
